# Obesity care knowledge and practice among primary care physicians in Klang valley: a cross-sectional study

**DOI:** 10.1186/s12875-025-02946-3

**Published:** 2025-08-18

**Authors:** Ganesh Sritheran Paneerselvam, Ling Siik Kee, Semira Abdi Beshir, Long Chiau Ming, Nada A. Alsaleh, Alaa A. Alsharif

**Affiliations:** 1 School of Pharmacy, Faculty of Health and Medical Sciences , Taylor’s University, Subang Jaya, Malaysia; 2 Department of Pharmacy Practice, Dubai Pharmacy College for Girls, Dubai, United Arab Emirates; 3 Datta Meghe College of Pharmacy, Datta Meghe Institute of Higher Education and Research (deemed to be University), Sawangi (M), Wardha, India; 4 Faculty of Medical and Life Sciences, Sunway University, Sunway City, Malaysia; 5 Department of Pharmacy Practice, College of Pharmacy, Princess Nourah Bint Abdulrahman University, P.O. Box 84428, Riyadh, 11671 Saudi Arabia

**Keywords:** Primary care physicians, Obesity, Knowledge, Practice

## Abstract

**Background:**

Obesity has become a public health concern as its prevalence has increased rapidly around the world, including Malaysia. Primary care physicians (PCPs) are the first point of contact for obese patients, playing a crucial role in managing obesity. This study aims to determine the level of obesity care knowledge and practices among PCPs and to identify factors associated with them.

**Methods:**

A cross-sectional study was conducted using a self-administered questionnaire distributed physically and online to PCPs. Pearson Chi-Square test was used to identify associations between demographic characteristics and obesity-related knowledge while the relationship between knowledge and practice scores was explored using regression analysis.

**Results:**

A total of 126 PCPs participated in the study. Of these, 72% demonstrated a good level of knowledge, and 66% showed good practices in obesity care. Use of clinical practice guidelines (CPGs) was significantly associated with higher knowledge scores, while both CPG use and prior training were significantly associated with better practice scores (*p* < 0.05). A moderate positive correlation was observed between knowledge and practice scores (*r* = 0.397, *p* < 0.001).

**Conclusion:**

PCPs have good obesity care knowledge and practice. Those using CPGs and with training were better prepared, resulting in improved practices. Enhancing awareness of these factors is crucial for future knowledge and practice improvements.

**Supplementary Information:**

The online version contains supplementary material available at 10.1186/s12875-025-02946-3.

## Introduction

Obesity is defined as the abnormal or excess adipose tissue accumulation which can pose a risk to health [1]. Its causes include the imbalance in energy expenditure and intake, genetic influences, behavioral factors, environmental factors, medical conditions, and unwanted side effects of the drugs [2]. It can be a significant risk factor for non-communicable diseases which can expose someone in having hypertension, coronary heart disease, sleep apnoea, fatty liver disease, type 2 diabetes mellitus, dyslipidaemia and so on. There are two main measures to determine obesity. The standard measure is through body mass index (BMI), with cut-off values of ≥ 23 kg/m² for pre-obesity and ≥ 27.5 kg/m² for obesity, and another important measure is waist circumference (WC), which defines obesity as ≥ 80 cm for women and ≥ 90 cm for men [3].

Obesity has emerged as a global health crisis, with its prevalence increasing at an alarming rate over the past few decades. In Malaysia, the situation is particularly concerning, with the country experiencing some of the highest rates of obesity in Southeast Asia. According to the Institute for Public Health (2024) [4], it was reported that around 54.4% of adults in Malaysia were overweight or obese, with 32.6% being overweight and 21.8% being obese. The trends of overweight and obesity among adults in Malaysia have increased from 50.1% in 2019 to 54.4% in 2023. Due to increased prevalence of obesity, primary care physicians (PCPs) should play a crucial role in the early identification, intervention, and management of obesity, making their knowledge and practice in this area vital to addressing the epidemic. Additionally, PCPs recognize that obesity is a chronic disease and a major healthcare concern, and they report having both the motivation and competence to discuss obesity issues and management with their patients [5]. In this regard, the importance of the Clinical Practice Guidelines for the Management of Obesity [3], to be utilized as a main reference source and referred by the PCPs in Malaysia. It provides evidence-based recommendations to the PCPs on the obesity management in Malaysia.

Studies have shown PCPs, may have negative attitudes towards obese patients, contributing to the low success rate of obesity management [6, 7]. Understanding physicians’ awareness is crucial for designing targeted training programs to address knowledge gaps. There is limited research assessing the knowledge and practices of PCPs on obesity care based on local CPG recommendations in Malaysia. Therefore, this study aims to determine the level of obesity care knowledge and practices among PCPs and to identify factors associated with them. The findings may offer valuable insights for future efforts to bridge the knowledge gap in obesity management, ultimately improving both healthcare providers’ proficiency and patients’ health outcomes.

## Materials and methods

### Study design

A cross-sectional study was conducted among PCPs in Klang Valley from February to May 2024. Using convenience and snowballing sampling method, the quantitative data was collected through a self-administered questionnaire distributed both physically and online.

### Sample size calculation

The estimated general population of PCPs in Klang Valley was around 459, derived from a reliable list of private primary care clinics participating in the Health Screening Program on the Social Security Organization website [8] and local state health department data. Using the Raosoft^®^ sample size [9] calculator, with 95% confidence level, 5% margin of error, and a response distribution of 50%, the estimated sample size required was 210.

### Study population

All PCPs around Klang Valley were approached to participate in this study. Only active general practitioners practicing at study area were included in the study while specialists or retires PCPs and not willing to provide consent were excluded from the study.

### Survey instrument

The survey instrument was developed by adapting methodologies from Martins and Norsett-Carr (2017) and the latest CPG for the Management of Obesity [3, 10]. It includes three sections: Section A gathers demographic information; Section B assesses PCPs’ knowledge of obesity management and Section C evaluates practice. Section B assesses PCPs’ knowledge of obesity management with 19 questions, with each correct response score of 1 point was given while 0 for incorrect response, yielding to a possible score of 0–19. While Section C evaluates obesity care practice using 5-point Linkert scale across 17 items, with a maximum score of 85. The median score from the total of each sections were used as a cut – off point to categorize both knowledge and practice levels. This method provides a simple and statistical valid method to split a continuous variable into two groups, facilitating comparison [11]. For Section B, knowledge, the median score obtained was 13, therefore sample scores 13 and above was classified into “good knowledge” and “poor knowledge” for sample with score less than 13. Similarly, for practice, a median score of 55 was used as a cut off point, resulting sample scores 55 and above categorized as “good practice” and score less than 55 as “bad practice”. This method of using median as cut off point was also applied in a previous study [12].

To ensure content validity, the questionnaire was reviewed by a panel of academic experts in pharmacy and clinical practice. Face validity was established through a pilot study among 21 PCPs who asses the clarity and comprehensiveness od the items. Based on their feedback, minor revision was made and internal consistency reliability was assessed using Cronbach’s alpha tests, yielding values of 0.71 for the knowledge section and 0.79 for the practice section, indicating good internal consistency.

### Data analysis

The collected data were analyzed using IBM SPSS Statistics version 29. Demographic information of the participants was expressed in frequency, percentage, n (%) and mean (standard deviation). While the continuous data for knowledge and obesity practices were summarized using median (IQR). The *p* value < 0.05 was considered statistically significant for all the tests. The Pearson Chi-Square test assessed the association between demographic characteristics and obesity-related knowledge and practice scores. Spearman’s rank correlation test evaluated the association between knowledge and practice scores, while regression analysis explored their overall relationship.

## Results

### Respondents’ demographic characteristics

The response rate was around 60% based on the targeted sample size 210. The low response rate was due to time constraints and a lack of interest among PCPs to participate in this study. The demographic characteristics of the respondents were summarized in Table 1. There was only a slightly higher number of male respondents (*n* = 67, 53.2%) with majority (*n* = 59, 46.8%) of respondents’ age were between 31 and 40 years of age with mean age (SD) of 42.1 (12.3) years old. A significant majority of respondents (72.2%) had not attended any training in obesity management. While most were aware of the Malaysian CPG for the management of obesity (65.1%), less than half (47.6%) reported using these guidelines in their practice.

**Table 1:** Demographic Characteristics of Respondents (*n*=126)

Variables	Number of Respondents *n* (%)	Mean (SD)
Gender		
Female	59 (46.8)	
Male	67 (53.2)	
Age		42.1 (12.3)
<30 years	14 (11.1)	
31-40 years	59 (46.8)	
41-50 years	24 (19)	
51-60 years	15 (11.9)	
>60 years	14 (11.1)	
Received obesity management training in the past one year		
Yes	35 (27.8)	
No	91 (72.2)	
Aware of the 2^nd^ edition (2023) of Malaysian Clinical Practice Guidelines for the Management of Obesity		
Yes	82 (65.1)	
No	44 (34.9)	
Use the 2^nd^ edition (2023) of Malaysian Clinical Practice Guidelines for the Management of Obesity as the source of information in your practice		
Yes	60 (47.6)	
No	66 (52.4)	

### Obesity care knowledge among PCPs

Most of the physicians (*n* = 72, 57.1%) scored above the median value 13. Majority of them were equipped with good knowledge, as summarized in Table 2. Conversely, high percentage of PCPs answered incorrectly pertaining to questions on minimum duration of moderate physical activity that should be recommended among obese patients, assessing the cost-effectiveness of a low-calorie diet alone versus a combination with orlistat and in identifying medications contribute to weight gain, with 74.6%, 61.9% and 70.6% respectively.

**Table 2:** Association between demographic characteristics and obesity care knowledge among PCPs

Items	Correct *n* (%)	False *n* (%)
1. Risk factors of obesity	94 (74.6)	32 (25.4)
2. Complications are associated with obesity	99 (78.6)	27 (21.4)
3. BMI can distinguish weight between muscle and fat.	102 (81)	24 (19)
4. The cut-off BMI value 23 kg/m² indicates the need to assess for overweight/obesity associated conditions.	89 (70.6)	37 (29.4)
5. The cut off waist circumference value of ≥90 cm for men and ≥80 cm for women indicates the need to assess for overweight/obesity associated conditions.	105 (83.3)	21 (16.7)
6. Goals of obesity management	106 (84.1)	20 (15.9)
7. It is realistic to aim for a 5–10% weight loss from baseline weight to improve the health of obese adults.	117 (92.9)	9 (7.1)
8. It is recommended to achieve at least 120 minutes per week of moderate intensity physical activity among all the obese individuals to have substantial health benefits.	32 (25.4)	94 (74.6)
9. It is recommended to achieve at least 75 minutes per week of vigorous intensity physical activity for weight loss among obese individuals.	80 (63.5)	46 (36.5)
10. It is recommended to have 300-1000 kcal/day deficit from daily energy requirement among the obese patients to improve the health outcome.	86 (68.3)	40 (31.7)
11. It is recommended to have 1200-1500kcal/day of energy intake for women and 1500-1800kcal/day for men to improve the health of obese adults.	90 (71.4)	36 (28.6)
12. Low-calorie diet is less cost effective compared to low-calorie diet and orlistat combination.	48 (38.1)	78 (61.9)
13. Combining diet, exercise, and cognitive behavioral therapy (CBT) is a less effective lifestyle treatment for obesity than diet and exercise combination.	95 (75.4)	31 (24.6)
14. The most likely contributor of weight gain after a period of weight loss is due to an increase in hunger sensation and a decrease in satiety caused by physiological adjustments to appetite and control systems.	100 (79.4)	26 (20.6)
15. Medications may contribute to weight gain.	37 (29.4)	89 (70.6)
16. Pharmacotherapy should be indicated for a patient with BMI ≥27 kg/m^2^ with weight-related medical comorbidities.	73 (57.9)	53 (42.1)
17. Pharmacotherapy should be indicated for a patient with BMI ≥30 kg/m^2^ with or without weight related medical comorbidities.	94 (74.6)	32 (25.4)
18. Bariatric Surgery should be considered for a patient with BMI ≥37.5 kg/m^2^ without any comorbidities to treat obesity.	78 (61.9)	48 (38.1)
19. Low levels of vitamin B12, vitamin D, calcium and iron are the common complications for the patient who underwent bariatric surgery.	102 (81)	24 (19)

### Obesity care practice pattern among PCPs

**Fig. 1:**
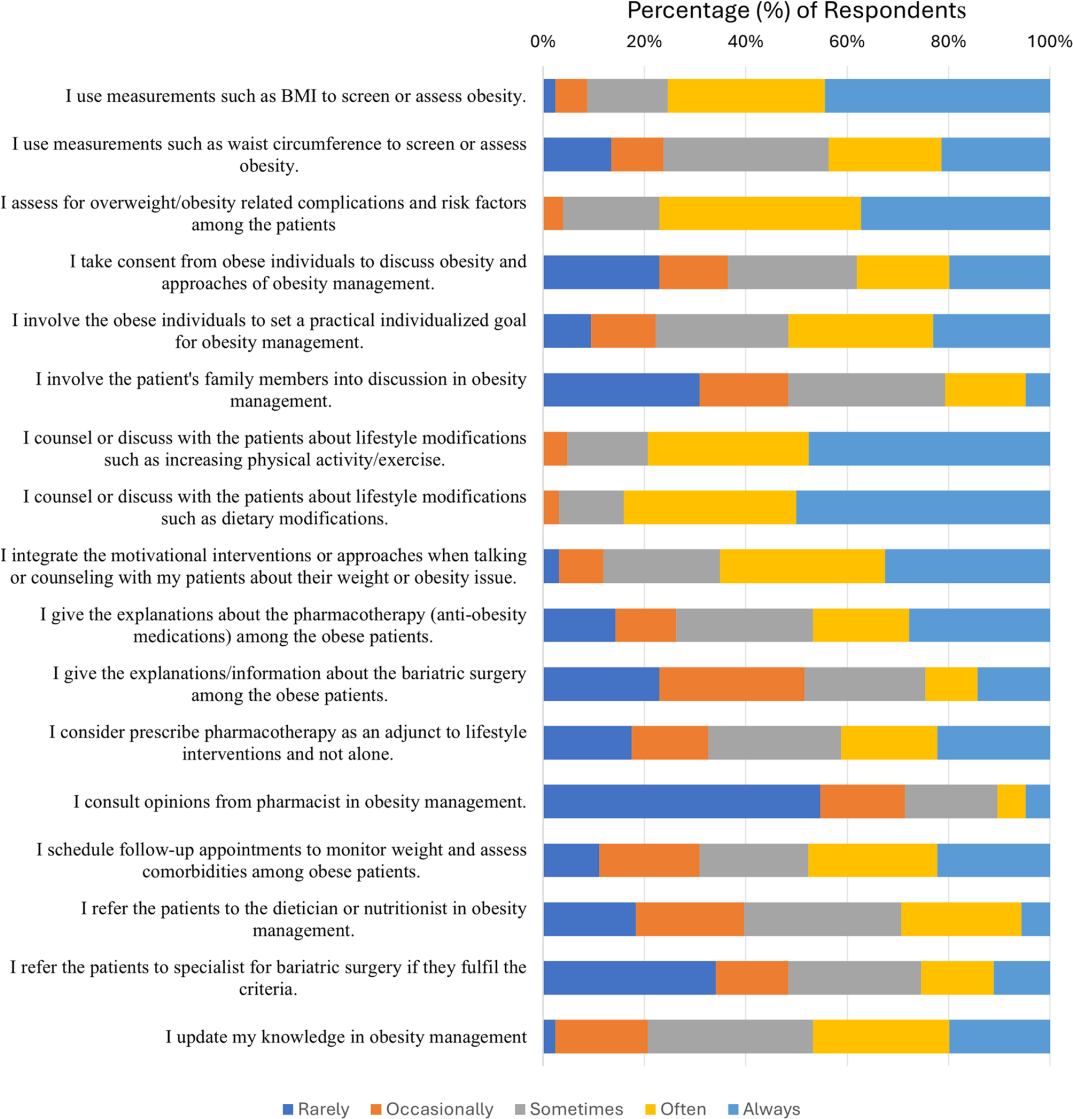
Obesity care practice pattern among PCPs

High proportion of respondents (*n* = 66, 52.4%) scored above the median score 55, indicating a good practice level. It was found that most of the respondents always use BMI to screen for obesity (*n* = 56, 44.4%) compared to using WC (*n* = 27, 21.4%). Unexpectedly, a very low proportion of respondents (*n* = 6, 4.8%) involve patient’s family members in the discussion of obesity management. When it comes to the involvement of pharmacists or referral to other health practitioners, low proportion of PCP’s practice it, (4.8% and 31%) (Fig. 1).

### Association between the demographic characteristics with obesity care knowledge and practice

**Fig. 2:**
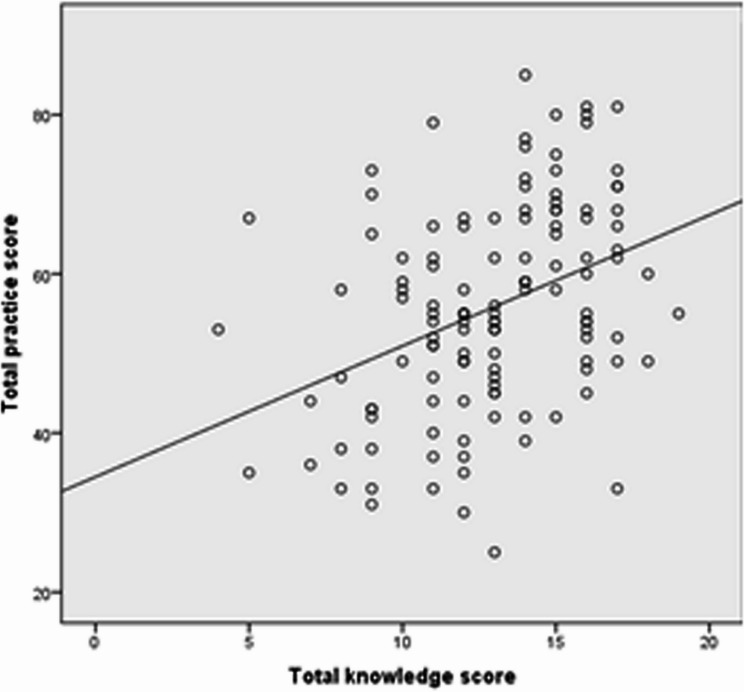
Relationship between obesity care knowledge and practice scores among primary care physicians

Awareness and use of Malaysian CPG for obesity management were significantly associated with better knowledge scores in obesity care (*p* < 0.05) (Tables 3 and 4). Additionally, respondents who received training in obesity management and used the Malaysian CPG demonstrated significantly improved practice skills (*p* < 0.05 (Fig. 2). This indicates that strong training and adherence to CPGs contribute to more effective obesity care practices and knowledge.

**Table 3:** Association between demographic characteristics and obesity care knowledge among PCPs

Variables	Obesity Care Knowledge *n* (%)		*P* value
	Poor	Good	
Gender			
Female	23 (18.3)	36 (28.6)	0.41
Male	31 (24.6)	36 (28.6)	
Age			
<30 years	9 (7.1)	5 (4)	0.337
31-40 years	26 (20.6)	33 (26.2)	
41-50 years	9 (7.1)	15 (11.9)	
51-60 years	4 (3.2)	11 (8.7)	
>60 years	6 (4.8)	8 (6.3)	
Received obesity management training in the past one year			
Yes	14 (11.1)	21 (16.7)	0.688
No	40 (31.7)	51 (40.5)	
Aware of the 2^nd^ edition (2023) of Malaysian Clinical Practice Guidelines for the Management of Obesity			
Yes	26 (20.6)	56 (44.4)	<0.001*
No	28 (22.2)	16 (12.7)	
Use the 2^nd^ edition (2023) of Malaysian Clinical Practice Guidelines for the Management of Obesity as the source of information in your practice			
Yes	17 (13.5)	43 (34.1)	0.002*
No	37 (29.4)	29 (23)

**Table 4:** Association between the demographic characteristics and obesity practice level among PCPs

Variables	Obesity Care Practice *n* (%)		*P* value
	Good	Poor	
Gender			
Female	25 (19.8)	34 (27)	0.269
Male	35 (27.8)	32 (25.4)	
Age			
<30 years	10 (7.9)	4 (3.2)	0.320
31-40 years	26 (20.6)	33 (26.2)	
41-50 years	9 (7.1)	15 (11.9)	
51-60 years	8 (6.3)	7 (5.6)	
>60 years	7 (5.6)	7 (5.6)	
Received obesity management training in the past one year			
Yes	8 (6.3)	27 (21.4)	<0.001*
No	52 (41.3)	39 (31)	
Aware of the 2^nd^ edition (2023) of Malaysian Clinical Practice Guidelines for the Management of Obesity			
Yes	27 (21.4)	55 (43.7)	<0.001*
No	33 (26.2)	11 (8.7)	
Use the 2^nd^ edition (2023) of Malaysian Clinical Practice Guidelines for the Management of Obesity as the source of information in your practice			
Yes	17 (13.5)	43 (34.1)	<0.001*
No	43 (34.1)	23 (18.3)	

### Correlation between obesity care knowledge score and practices score among PCPs

Spearman’s rank correlation test shows positive correlation between the knowledge and practice scores, which was statistically significant (*r* = 0.397, *p* < 0.001). Through regression model, the following equation was obtained:

obesity practice score = 34.478 + 1.65 (obesity knowledge score). This indicated for every unit increase in knowledge, obesity care practices were expected to increase by 1.65 units.

## Discussion

### Obesity care knowledge of primary care physicians

Most of the PCPs demonstrated a good level of knowledge regarding obesity care. However, a significant proportion of PCPs not aware the minimum duration of moderate intensity exercise needed for obese patients. This finding was consistent with another study that indicated poor recommendation of physical activity by PCPs [13]. This could be due to a lack on training on the physical exercise aspect resulting in unawareness of the specific amount or duration of exercise required to maintain and improve the health of obese patients [14]. Besides, most of the respondents believed that low calorie diet was less cost-effective compared to the combination with orlistat. According to study by van Baal et al. (2008), using incremental cost per QALY gained suggested that combination of orlistat plus low caloric diet was cost ineffective interventions compared to low caloric diet al.one [15]. The knowledge gap may be attributed to that those physicians were lack of knowledge, training on health economics or pharmacoeconomic aspects [16].

Moreover, most respondents unable to identify medications that contributes to weight gain. This may be because respondents did not have adequate knowledge about obesogenic medications and limited collaboration between pharmacists due to lack of time and compensation [17]. It is important to increase knowledge about this aspect because patients who took obesogenic medications were more likely to fail to achieve weight loss of 5% and resulted in a poor weight reduction outcome [18].

### Obesity care practices among primary care physicians

Most of the PCPs in our study preferred using BMI to screen or assess obesity, as in line with another study [19]. This preference was due to BMI’s quick result, non-intrusive nature, and convenience for screening and measuring weight categories [20]. PCPs are encouraged to use waist circumference to estimate central obesity in Asian populations, as BMI often underestimates excess adiposity and its complications [21].

PCPs should involve family members in discussions for support in obesity management and lifestyle changes [5]. PCPs seldom refer obese patients to other professionals due to unfamiliarity with the importance of such referrals [22]. They rarely consult pharmacists in obesity management because many practitioners in Malaysia run solo practices and perceive increased costs, lack confidence in pharmacists, and are uncertain about their roles [23]. A multidisciplinary approach has been shown to improve self-efficacy and weight loss outcomes [24]. Future initiatives should focus on helping PCPs develop and integrate multidisciplinary approaches in obesity management.

### Factors associated with obesity, care, knowledge and practices among PCPs

The study found that awareness and use of the Malaysian CPG for Obesity Management were linked to better knowledge and practices among PCPs. Low CPG knowledge among PCPs leads to poor adherence to guidelines and inadequate use of intensive behavioral therapy and pharmacotherapy for obese patients [25]. Training in obesity management had a more significant impact on practices than knowledge, likely due to varying training quality [26]. Such training improved healthcare providers’ ability to manage weight issues, motivate patients, and gain confidence [27]. Our study shows a positive correlation between knowledge and practices, consistent with Carrasco et al. (2022) [20], which states that physicians achieved knowledge internalization through expert information, professional websites, and educational training enhances physicians’ competence and confidence, improving obesity treatment.

Despite showing moderate strength, there may be other possible factors which might influence the practice of obesity care among PCPs. Previous literature has identified a few barriers that hinder optimal obesity management by PCP which includes inadequate training for nutritional therapy (which empowers physician with diet management plans for obese patients), limited consultation time due to heavy workload and lack of reimbursement for obesity related programs. Moreover, lack of institutional support in establishing a multidisciplinary care team and organizing specialized training in obesity medicine periodically, further contributes to the barrier to PCP practice.

### Strength and limitation

This study offers a few strengths which enrich the physician discipline on obesity management in primary settings. One key strength is the focus on both knowledge and practice of PCP, providing a more comprehensive view on the gaps between awareness and clinical application. Unlike other studies that focus on clinical outcomes, this study integrates local clinical guideline into the design of the questionnaire ensuring contextual relevance and application to Malaysian healthcare landscape. Lastly, this study informs the importance in training and policy development in strengthening obesity care delivery. However, this study has several limitations. Firstly, self-reported measures by the PCP for assessing knowledge and practice may introduce social desirability bias, as physicians may have overestimated their actual knowledge or practice that aligns with perceived expectation rather than routine behavior. Secondly, as the study was done in a single geographic region, the results may affect the generalizability of the findings to other countries. Future research may be conducted in broader geographic regions and examining other possible stakeholders to provide a more comprehensive understanding of obesity care among PCPs.

## Conclusion

This study identifies gaps in PCPs’ knowledge and practices, especially regarding physical exercise, pharmacoeconomics, and family involvement in obesity care. Key factors for good knowledge and practice include awareness and use of updated CPG and recent obesity management training. Future efforts should focus on enhancing PCP education and resources to improve obesity care and patient outcomes.

## Supplementary Information

Supplementary Material 1.

## Data Availability

All data have been presented in the manuscript.

## Abbreviations

PCPs: Primary Care Physicians
CPGs: Clinical Practice Guidelines
BMI: Body Mass Index
WC: Waist Circumference
QALY: Quality-Adjusted Life Year
SPSS: Statistical Package for the Social Sciences
SD: Standard Deviation
