# Supplementary material for: Obesity care knowledge and practice among primary care physicians in Klang valley: a cross-sectional study
Source: BMC Prim Care. 2025 Aug 18;26:256. doi: 10.1186/s12875-025-02946-3 (PMC12359962; doi:10.1186/s12875-025-02946-3)
Supplement: Supplementary file 1 — Supplementary Material 1. [file 12875_2025_2946_MOESM1_ESM.pdf]

APPENDICES

Appendix 1: Questionnaire

Section A Demographic information

Kindly tick (√) the information that best describes you:

1) Gender

□Female
□Male

2) Ethnicity
□Malay
□Chinese
□Indian
□Others, please specify:________________________

3) Age

______________years old

4) Years of Practice

______________years

5) Current healthcare sector
□Public
□Private

6) Profession/Health discipline
□General Practitioner
□Specialist in Family Medicine or Internal medicine
□Others: Specify ____________________

7) Number of obese patients seen in a week at the practice setting?
□<5
□5-10
□>10

8) Have you received obesity management training in the past one year?
□Yes
□No

9) Are you aware of the 2nd edition (2023) of Malaysian Clinical Practice Guidelines for the Management of Obesity?
□Yes
□No

Section B - Questions on Knowledge
Kindly tick (√) the option (s) that is/are applicable.
1) Which of the following is/are the risk factors of obesity? (Select option(s) that is/are applicable)
□Lack of physical activity
□Unhealthy eating behaviour
□High alcohol intake
□Lack of sleep
□High amount of stress
□All of the above

2) Which of the following complications are associated with obesity? (Select option(s) that is/are applicable)
□Type 2 Diabetes Mellitus
□Hypertension
□Osteoarthritis
□Obstructive sleep apnoea
□Reproductive infertility
□Stress incontinence
□Depression
□All of the above

3) BMI can distinguish weight between muscle and fat.
□True
□False
□Not sure

4) The cut-off BMI value 23 kg/m² indicates the need to assess for overweight/obesity associated conditions.
□True
□False
□Not sure

5) The cut off waist circumference value of ≥90 cm for men and ≥80 cm for women indicates the need to assess for overweight /obesity associated conditions.
□True
□False
□Not sure

6) What are the goals of obesity management? (Select option(s) that are applicable)
□Prevent the obesity related complications.
□Prevent or treat the comorbidities.
□Combat against the weight stigma
□Reestablish one's well-being and self-esteem
□All of the above

7) It is realistic to aim for a 5–10% weight loss from baseline weight to improve the health of obese adults.
□True
□False
□Not sure

8) It is recommended to achieve at least 120 minutes per week of moderate intensity physical activity among all the obese individuals to have substantial health benefits.
□True
□False
□Not sure

9) It is recommended to achieve at least 75 minutes per week of vigorous intensity physical activity for weight loss among the obese individuals.
□True
□False
□ Not sure

10) It is recommended to have 300-1000 kcal/day deficit from daily energy requirement among the obese patients to improve the health outcome.
□True
□False
□Not sure

11) It is recommended to have 1200-1500kcal/day of energy intake for women and 1500-1800kcal/day for men to improve the health of obese adults.
□True
□False
□Not sure

12) Low-calorie diet is less cost effective compared to low-calorie diet and orlistat combination.
□True
□False
□Not sure

13) Combining diet, exercise, and cognitive behavioral therapy (CBT) is a less effective lifestyle treatment for obesity than diet and exercise combination.
□True

☐False

☐Not sure

14) The most likely contributor of weight gain after a period of weight loss is due to an increase in hunger sensation and a decrease in satiety caused by physiological adjustments to appetite and control systems.

☐True

☐False

☐Not sure

15) Which of the following medications may contribute to weight gain?
(Select option(s) that is/are applicable)

☐Insulin

☐Propranolol

☐Selective serotonin reuptake inhibitors (SSRI)

☐Prednisolone

☐Cetirizine

☐All of the above

16) Pharmacotherapy should be indicated for a patient with BMI ≥27 kg/m^2 with weight-related medical comorbidities.

☐True

☐False

☐Not sure

17) Pharmacotherapy should be indicated for a patient with BMI ≥30 kg/m^2 with or without weight related medical comorbidities.

☐True

☐False

☐Not sure

18) Bariatric Surgery should be considered for a patient with BMI ≥37.5 kg/m^2 without any comorbidities in order to treat the obesity.

☐True

☐False

☐Not sure

19) Low levels of vitamin B12, vitamin D, calcium and iron are the common complications for the patient who underwent bariatric surgery.

☐True

☐False

☐Not sure

Section C - Questions on Obesity Practice

Kindly tick (√) the option that is most applicable to you.

1) Do you use the 2nd edition (2023) of Malaysian Clinical Practice Guidelines for the Management of Obesity as the source of information in your practice?

- Yes
- No

2) Below shows the statements about obesity practice. For each statement, select/circle one response which is most applicable to you.

(Scale 1-5; where 1=Rarely, 2= Occasionally, 3= Sometimes, 4= Often, 5=Always.

	Obesity care practice	Rarely	Occasionally	Sometimes	Often	Always
1	I use measurements such as BMI to screen or assess obesity.	1	2	3	4	5
2	I use measurements such as waist circumference to screen or assess obesity.	1	2	3	4	5
3	I assess for overweight/obesity related complications and risk factors among the patients.	1	2	3	4	5
4	I take consent from obese individuals to discuss obesity and approaches of obesity management.	1	2	3	4	5
5	I involve the obese individuals to set a practical individualized goal for obesity management.	1	2	3	4	5
6	I involve the patient's family members into discussion in obesity management.	1	2	3	4	5
7	I counsel or discuss with the obese patient about lifestyle modifications such as increasing physical activity/exercise.	1	2	3	4	5

8	I counsel or discuss with obese patients about lifestyle modifications such as dietary modification.	1	2	3	4	5
9	I integrate the motivational interventions or approaches when talking or counseling with my patients about their weight or obesity issue.	1	2	3	4	5
10	I give the explanations about the pharmacotherapy (anti-obesity medications) among the obese patients.	1	2	3	4	5
11	I give the explanations/information about the bariatric surgery among the obese patients.	1	2	3	4	5
12	I consider prescribe pharmacotherapy as an adjunct to lifestyle interventions and not alone.	1	2	3	4	5
13	I consult opinions from pharmacist in obesity management.	1	2	3	4	5
14	I schedule follow-up appointments to monitor weight and assess comorbidities among obese patients.	1	2	3	4	5
15	I refer the patients to the dietician or nutritionist in obesity management.	1	2	3	4	5
16	I refer the patients to specialist for bariatric surgery if they fulfil the criteria.	1	2	3	4	5
17	I update my knowledge in obesity management.	1	2	3	4	5
